# Clinical characterization of 266 patients and family members with cleft lip and/or palate with associated malformations and syndromes

**DOI:** 10.1007/s00784-021-03863-2

**Published:** 2021-03-24

**Authors:** Theodosia Bartzela, Björn Theuerkauf, Elisabeth Reichardt, Malte Spielmann, Charlotte Opitz

**Affiliations:** 1 Institute of Dental and Craniofacial Sciences, Dept. of Orthodontics, Dentofacial Orthopedics, and Pedodontics, Charité – Universitätsmedizin Berlin, corporate member of Freie Universität Berlin, Humboldt-Universität zu Berlin, and Berlin Institute of Health, Aßmannshauser Str. 4-6, 14197 Berlin, Germany; 2 Ringpromenade 76, 14612 Falkensee, Berlin, Germany; 3 Klinik für Pediatric Oral Health und Kieferorthopädie, Universitäres Zentrum für Zahnmedizin Basel, Mattenstrasse 40, 4058 Basel, Switzerland; 4 Human Molecular Genomics Group, Max Planck Institute for Molecular Genetics, 13353 Berlin, Germany; 5 Institute of Human Genetics, University of Lübeck, Lübeck, Germany

**Keywords:** Cleft lip and palate, Associated anomalies, Syndromes, Genetics, Pedigrees

## Abstract

**Objectives:**

To clinically characterize patients and family members with cleft lip and/or palate (CL/P) and associated congenital malformations or syndromes and propose possible inheritance patterns.

**Materials and methods:**

An observational study of patients with CL/P, including medical and family history and intra- and extra-oral examination of their family members, was performed.

**Results:**

Two hundred sixty-six patients, 1257 family members, and 42 pedigrees were included in the study. The distribution of patients according to the cleft type was 57.9% with CLP, 25.2% with cleft palate (CPO), and 12.8% with cleft lip with/without alveolus (CL/A). Seventy-four (27.8%) patients had associated malformations, and 24 (9.2%) a syndrome. The skeletal (27.7%), cardiovascular (19.3%) systems, and eyes (22.9%) were most commonly affected. Pierre Robin Sequence (7 patients) and van der Woude (4) were the most common syndromes. The majority of patients with CPO (19/24) had an associate syndrome. The families had an average of 2.45 affected members.

**Conclusion:**

Individual and interfamilial phenotypic variability in patients with CL/P makes the understanding of etiopathogenesis challenging.

**Clinical relevance:**

The overall prevalence of individuals with CL/P and their pedigrees with associated malformations and syndromes emphasize the need for early identification, interdisciplinary, and long-term planning.

**Supplementary Information:**

The online version contains supplementary material available at 10.1007/s00784-021-03863-2.

## Introduction

Cleft lip and/or cleft palate (CL/P) is the most common orofacial deformity. It is classified among the major structural anomalies that have a significant medical and social impact on the affected individuals and their families [1]. The prevalence reported worldwide is 5.44 per 10,000 live births [1], but it varies depending on ethnicity, geographical background, phenotypic severity, and socioeconomic conditions [2]. Even though the majority of orofacial clefts (OFC) (almost 70%) are isolated or nonsyndromic (nsCLP) [3], there are types, like the cleft palate (CPO), that are more often associated with a syndrome (50%) [4] or a congenital malformation (46.7%) in comparison to CLP (36.8%) or cleft lip (CL) (13.6%) phenotypes [5]. Patients with CPO, though, have a less common (22%) positive family history for an OFC in comparison to patients with cleft lip and alveolus (CLA) (26%) or CLP (29%) [6]. Nevertheless, the severity of cleft seems to play a role in the association with a syndrome or a malformation. Thus, patients with bilateral CLP (BCLP) have more frequent associated congenital malformations or syndromes in comparison to patients with unilateral CLP (UCLP) [7]. The reported prevalence of these abnormalities is ranging between 3 and 63.4% [8]. Various associated anomalies have been monitored mainly in craniofacial (68% in the mandible) and neck area [6], but also the cardiovascular (24–51%), gastrointestinal and urogenital systems, in the skeleton (especially in vertebral column) and extremities, and the ears and eyes [5]. Hence, more than 600 syndromes have been identified with OFC [9], of which more than 400 have been associated with CPO. The etiopathogenesis of nsCLP from syndromic CLP (sCLP) patients has been differentiated. The family history of nsCLP has a different degree of recurrence depending on the cleft’s type and severity [10]. Even though the family recurrence in patients with a CL/P is more often than in other congenital malformations [10], and the involved genes and the time frame of cleft occurrence are defined, the molecular basis remains unclarified [11]. Genetics plays a significant role in the etiopathogenesis of nsCLP, but still, differential environmental factors overlap, as it has been proved in monozygotic twins (MZ) [4, 11]. Several exogenous factors such as race, sex, parental age [12], maternal exposure to teratogenic agents or medication, poor nutrition, alcohol, smoking, or viral infections the first three months of pregnancy [13] have been investigated for the etiopathogenesis and the phenotypic expression of OFC. Familial recurrence patterns of CL/P have been reanalyzed from several family studies [14]. Moreover, the associated anomalies and syndromes have been a concern since they have been underdiagnosed in the first months or even years of a child’s life [15].

Therefore, in this study, we aim to present the clinical phenotype of the investigated 266 patients with CL/P with associated anomalies or syndromes and their family members. Furthermore, to show the inheritance patterns as it has been extracted from the included pedigrees.

## Materials and methods

Ethical approval was obtained from the Charité - Universitätsmedizin Berlin, where the study was conducted. Interdisciplinary treatment for OFC patients has been established by the Department of Oral and Maxillofacial Surgery and the Department of Orthodontics Dentofacial Orthopedics since 1965. Interdisciplinary consultation sessions are offered for these children regularly. The ethical principles of the Declaration of Helsinki for research on human subjects have been respected.

### Patients and their pedigree

All patients examined between 28.01.1999 and 25.05.2000, were recruited. Data of 603 patients were scrutinized. Patients were asked to participate in the study, together with their family members. From the contacted persons, only 266 index patients with CL/P had accepted to participate. Patients with submucosal clefts, non-Caucasian ethnic background, or adopted were excluded. The patients’ pedigree was traced by a personal interview with the family members (Supplementary information (SI 1)) and compared to the patient’s file. All available families were recruited for the construction of the pedigrees. The number of generations presented in each pedigree depended on the participated family members.

Stillbirths, neonatal deaths, and relatives’ deaths with CL/P and associated anomalies or syndromes were inconsistently reported from the family members. Therefore, this information was registered only in isolated cases. All patients and family members signed informed consent for participation in the study.

### Methods

The cleft’s type and severity, associated malformations, and syndromes were extracted from patients’ electronic files. Classification of the cleft was based on the method described by Koch et al. [16] according to the anatomical involvement as follows: the cleft lip (CL) with or without alveolus (CL/A), cleft lip and palate (CLP), cleft palate only (hard and soft) (CPO), and cleft soft palate only (CSO). The sidedness (right, left), the laterality (unilateral, bilateral), and the severity (complete, incomplete) were also encompassed. The anatomical cleft involvement has been presented in this study and not the palatal cleft’s severity based on the Arabic numerals according to the classification of Koch et al. [16]. The reason is that this information was often uncertain if the participants have been operated in other cleft centers or under the nonstandardized surgical procedure.

Two dentists of the cleft team performed intra- and extra-oral examination for index patients and their relatives [17] in the two dental chairs used for the interdisciplinary team consultation. The patients’ medical and family history was supplemented by a standardized questionnaire used by the Department of Human Genetics of Charité - Universitätsmedizin, adapted to the study’s needs (SI 1). The questionnaire was fully answered by the patients. In case of a suspected syndrome, the diagnosis was confirmed by a clinical geneticist. Additional data were collected from the Institute of Medical and Human Genetics and the Department of Audiology and Phoniatrics of Charité - Universitätsmedizin.

### Statistical analysis

Patients’ personal data were concealed in code, and data were analyzed by Microsoft Excel 97. The chart representation was performed in Excel 4.0 (Microsoft) and transferred to SPSS 9.0 Windows (Microsoft). For the exact pedigree representation, Cyrillic 2 and 2.1 were used.

Pearson chi-square test was used to evaluate the distribution of the cleft type in males and females. Fischer’s exact test was employed to assess the relationship between the cleft type’s prevalence with the cleft laterality and the association to a syndrome or a malformation.

## Results

### Index patients and CL/P family history

Files of 603 patients with CL/P were scrutinized for eligibility. Microforms of clefts or associated anomalies were detected in 161 individuals. Overall, 266 patients (from 263 families, including three siblings) were willing to participate in the study.

The participants’ distribution, according to the cleft type, laterality, sidedness, and sex, is presented in Table 1. The distribution of the cleft phenotypes of the index patients is as follows: 154 (57.9%) with CLP, 34 (12.8%) with CL/A, 67 (25.2%) with CPO, and 11 (4.1%) with other types of cleft (Table 1). Patients with a positive family history were distributed as follows: 44 (69.8%) individuals with CLP, 10 (15.9%) with CL/A, eight (12.7%) with CPO, and one (1.6%) with a left-sided CLA and cleft of the soft palate (CSO) (Table 1).

**Table 1:** Distribution of index patients (absolute and percentage (%)) according to cleft type, sex, and laterality of CL/P

Type of cleft	Patients *N* (%)		Male *N* (%)		Female *N* (%)		Right *N* (%)		Left *N* (%)		Bilateral *N* (%)		Syndromes
	Index patients	Index patients with PFH nonsyndromic	Index patients	Patients with PFH	Index patients	Patients with PFH	Index patients	Patients with PFH	Index patients	Index patients with PFH	Index patients	Index patients with PFH	
CLP	154 (57.9%)	44 (16.5%)	110 (41.5%)	38 (14.3%)	44 (16.5%)	17 (6.4%)	25 (9.3%)	7 (0.9%)	77 (28.9%)	21 (7.9%)	52 (19.5%)	16 (6.0%)	3 (12.5%)
CL/A	34 (12.8%)	10 (3.7%)	16 (6.0%)	0	18 (6.8%)	0	7 (2.6%)	2 (0.7%)	21 (7.9%)	7 (0.9%)	6 (2.2%)	1 (0.4%)	1 unilateral + 1 bilateral (CL/A) (8.4%)
CPO or CSO	67 (25.2%)	8 (3.0%)	27 (10.1%)	1 (0.4%)	40 (15.0%)	7 (0.9%)	NA	0	NA	0	NA	0	16 CPO (66.6%) + 3 CSO (12.5%)
Other type of cleft	11 (4.1%)	1 (0.4%)	7 (2.6%)	0	4 (1.5%)	0	NA	0	NA	0	NA	0	
Total patients	266 (100%)	63 (23.6%)	160 (60.2%)	39 (14.7%)	106 (39.8%)	24 (9.0%)	32 (12.0%)	9 (3.4%)	98 (36.8%)	28 (10.5%)	58 (21.8%)	17 (6.8%)	24 (9.2%)
Families	263	62 (26.6%)											22 (8.4%)

*CLP*, cleft lip and palate; *CL/A*, cleft lip with or without alveolus; *CPO*, cleft palate only; *CSO*, cleft of the soft palate only; *N*, number of patients; *NA*, not applicable; *PFH*, patients with positive family history among index patients

**Fig. 1:**
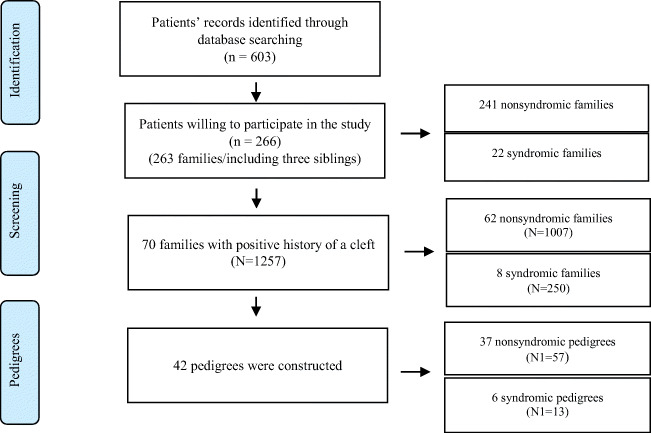
Patients’ distribution according to the initial patients’ pool. Patients’ family members were recruited for the development of the pedigrees. *n*, number of patients with a cleft; *N*, affected and non-affected family members; *N*1, family members with a cleft lip and/or palate

Positive family history was reported in 70 (26.6%) out of the 263 families. Among these families, 62 had nonsyndromic members (nsCL/P), and eight had at least one member with a syndrome. In total, 1257 family members participated in the study. Among them, 1007 were nonsyndromic and 250 syndromic (Fig. 1).

The 62 nonsyndromic families had 90 afflicted members, excluding the index patients (Table 2). Conclusively, the families had an average of 2.45 affected individuals (including the index patients).

**Table 2:** Affected family members of nonsyndromic and syndromic cleft patients

Affected family members of nonsyndromic patients	Number of families of nonsyndromic patients	Affected family members of syndromic patients	Number of families of syndromic patients
0	179	0	16
1	44	1	2
2	12	2	1
3	3	3	2
4	2	4	0
5	1	5	1
Total 90	241	15	22

The nsCL/P group of index patients encompassed 29 paternal relatives, 25 maternal, and four from both sides, maternal and paternal afflicted (Table 3). Moreover, Table 3 shows that CL/P was the most common type of cleft in the maternal and paternal relatives. Among the affected first-degree relatives were 15 (6.2%) parents, five (2.1%) siblings, and in one family were one parent and one sibling (0.8%). Affected family members were distributed according to their cleft type in CL/A (29.4%), CL/P (28.6%), and CPO (11.9%). More maternal lineage relatives were afflicted with CPO than paternal (5 over 2) (Table 3).

**Table 3:** Type of cleft distribution of nonsyndromic patients of the affected family members. Differentiated maternal and paternal relatives (62 out of the 241 nonsyndromic families)

Type of cleft	Maternal relatives	Paternal relatives	Maternal and paternal relatives
CLP	16	21	4
CL/A	4	6	0
CPO	5	2	0
Total	25	29	4

*CLP*, cleft lip and palate; *CL/A*, cleft lip with or without alveolus; *CPO*, cleft palate only

Altogether, in this study, there is a predominance of the affected mothers (ten mothers) in comparison to fathers (six). In this study, none of the index patients had both parents afflicted with a CL/P.

### Pedigrees

The families with affected individuals willing to participate in the study were all included. From these families, 42 pedigrees were constructed (SI 2). We were able to recruit more than four generations in 36 pedigrees (SI 2). In total, the nonsyndromic pedigrees were 37, and the syndromic were six. On average, 1.6 affected persons per family were registered. The nonsyndromic families had 57 members afflicted with a cleft (1.5 affected individuals per family), and the syndromic families had 13 members with an OFC (2.2 members on average).

### Cleft type, sidedness, laterality, and sex distribution

In this study, 98 (36.8%) out of the 266 index patients had a left-sided cleft, 32 (12%) a right-sided, 58 (21.8%) BCLP, 67 (25.1%) CPO, and 11 (4.1%) other types of clefts. Only one patient had a median upper lip cleft (0.4%) (Table 1). Unilateral clefts are predominant, representing 65.7% of the included patients (Table 1).

Of the 266 patients included, 160 (60.2%) were males, and 106 (39.8%) were females. It was observed a sex predisposition according to the cleft type (one-sided Fisher’s exact, *p* < 0.01). Therefore, in CLP phenotype, males were (71.4%) more commonly affected (28.6%), and in CPO, females had a predominance (females: 59.7%, males: 40.3%) (Table 1). Patients with CL/A (females: 52.9%, males: 47.1%) were almost equally distributed. Nearly 24% of the index patients had a positive family history, and most of them were males (males: 61.9% and females 38.1%) (Table 1).

In our data, it seems like there is a higher risk of recurrence of CL/P in the male offsprings of affected mothers. Furthermore, affected mothers also had offsprings afflicted with CPO of two different families.

Contrarily, in patients with CLA, the affected fathers were more (three) than the mothers (one). In total, the affected family members were more in the paternal lineage than the maternal (20 paternal and 16 maternal) (Table 3). There was a significant association with the patients’ cleft type, sex, and positive family history (*p* < 0.01). The cleft type had a significant relation with the cleft laterality and the association with a syndrome or a malformation (*p* < 0.001).

### Associated congenital malformations

**Fig. 2:**
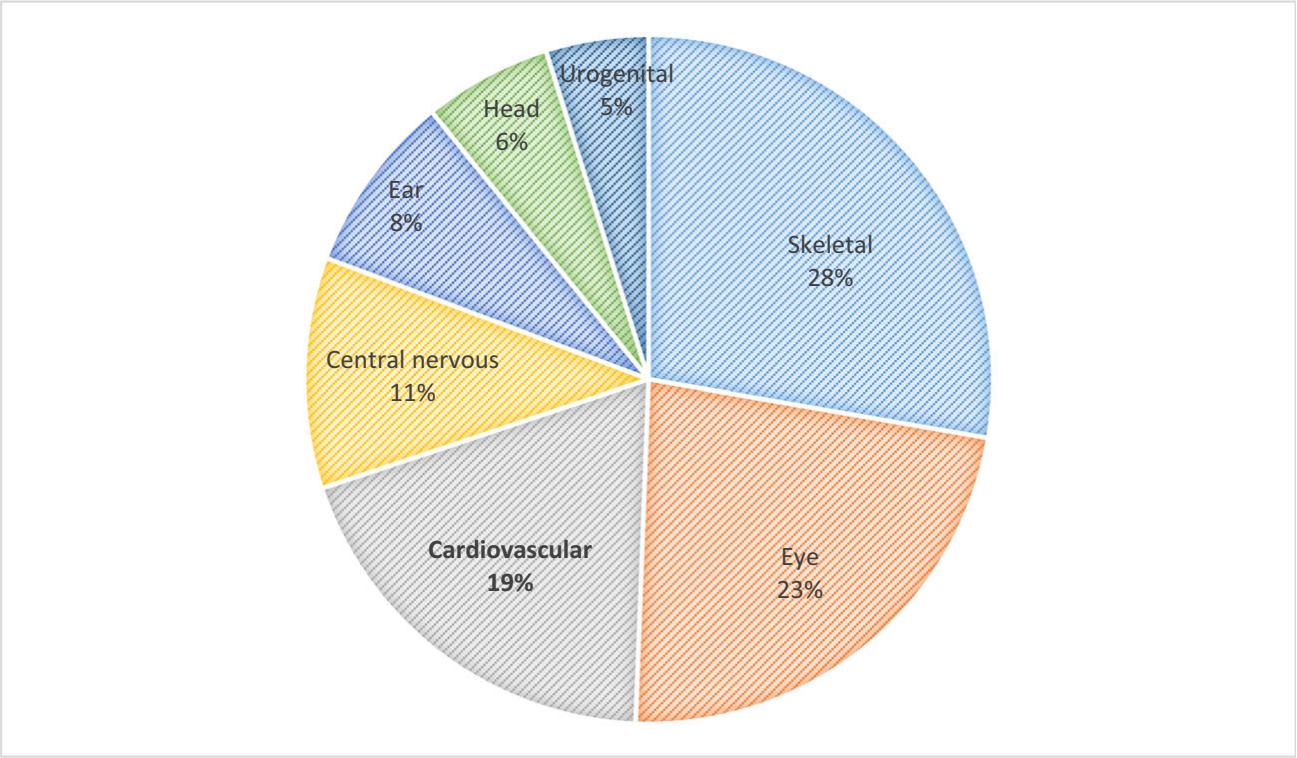
Distribution (%) of the associated malformations (83 malformations) in 55 of the 266 index patients

Among the 266 index patients with OFC, 27.8% had an associated malformation. The 242 nonsyndromic index patients’ examination revealed 55 (22.7%) members with at least one associated malformation (Fig. 2). In total, 83 congenital abnormalities have been detected in these 55 patients (Fig. 2). The most frequently reported malformations in this group of patients were of the skeletal system (in hands, feet, legs, vertebral column, and thorax) (27.7%) and eyes (22.9%), followed by cardiovascular (cardiac valve, septum, and the conduction system) (19.3%) and of the central nervous system (CNS) (10.9%) (encephalic abnormalities and profound mental and motoric retardation) (Fig. 2).

### Associated syndromes

Within the 266 index patients, 24 (9.2%) patients had an associated syndrome or sequence (Table 1). Among them, seven were diagnosed with Pierre Robin Sequence (PRS), five with van der Woude syndrome (VWS), three with Stickler syndrome, three with craniofacial dysmorphia (only clinical diagnosis available), two with Nager syndrome, and unique patients with Franceschetti syndrome, Kabuki syndrome, partial trisomy of chromosome 13, and translocation of chromosomes 7 and 14 (Table 4).

**Table 4:** Syndromes and chromosomal disorders in 266 patients with cleft lip and/or palate

Syndromes	Number of patients (*n*)	Percentage (out of the 266 index patients)
Pierre Robin Sequence	7	2.6%
van der Woude syndrome	5 (in 1 patient, the syndrome was suspected)	1.9%
Stickler syndrome	3 (two are siblings)	1.1%
Facial/craniofacial dysmorphia	3	1.1%
Nager syndrome	2 (siblings)	0.7%
Franceschetti syndrome	1	0.4%
Kabuki syndrome	1	0.4%
Trisomy 13 (partial)	1	0.4%
Translocation of chromosomes 7 and 14	1	0.4%
Total	24	9.2%

**Fig. 3:**
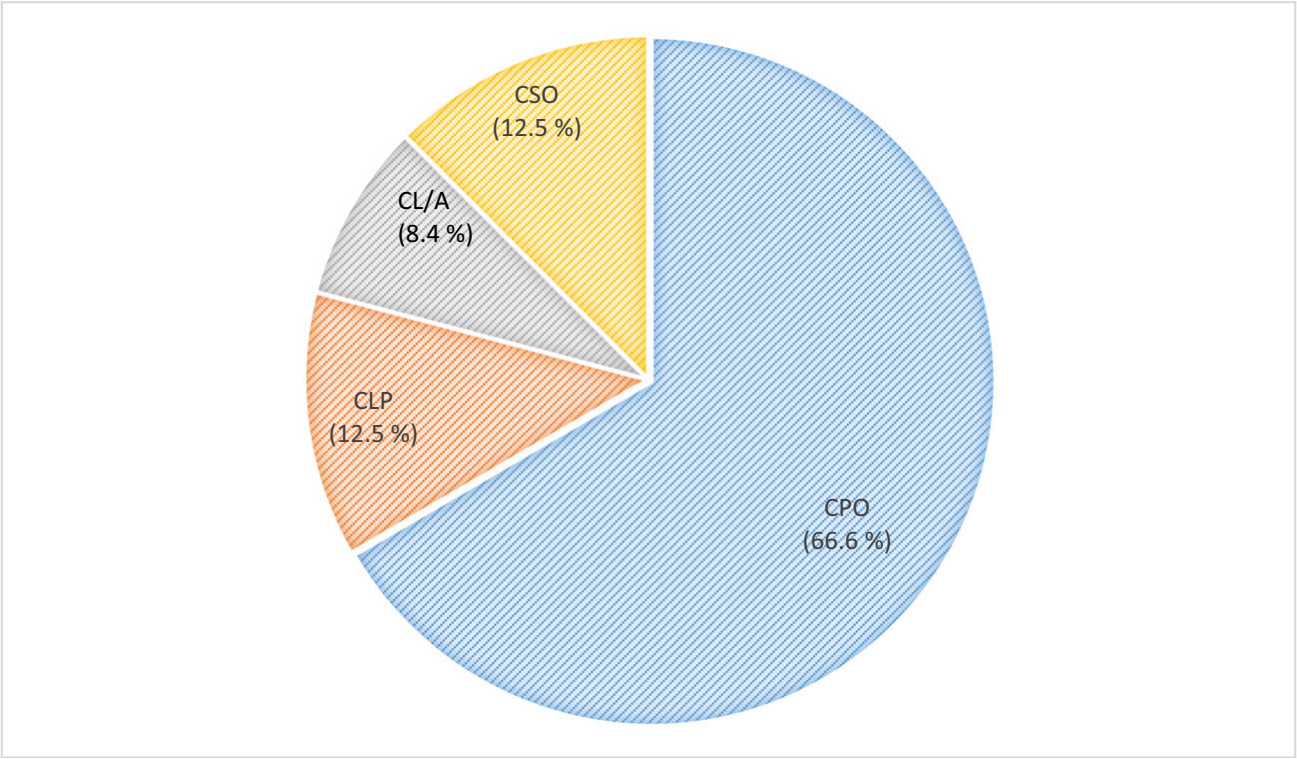
Patients with an associated syndrome according to the type of cleft (CL/A, cleft lip with or without alveolus; CLP, cleft lip and palate; CPO, cleft palate only (hard and soft); CSO, cleft soft palate only)

According to sex distribution, 13 were males and 11 females. Two of them were siblings. In total, 22 (8.7%) out of the 43 pedigrees had at least one family member with an associated syndrome. The syndromic patients had the following distribution according to their cleft type: 16 with CPO, three with CLP, three with a cleft of the soft palate (CSO), one with CLA and soft palate (CSO), and one with CL/A (Table 1). Almost 70% of the syndromic patients had a CPO (Fig. 3).

### Inheritance

At least four generations were recorded in more than 85% of the pedigrees (SI 2).

There is strong evidence of autosomal dominant (AD) transmission observed in three sequential generations, even with minor symptoms, in five of these pedigrees (SI2 pedigrees 2, 23, 28, 32, 40).

AD inheritance trait was presumed for 16 pedigrees observed in two sequential generations (pedigrees 3, 6, 7, 12, 15, 16, 17, 20, 21, 25, 27, 31, 33, 37, 38, 39). An (autosomal recessive) AR-pattern is suspected in three pedigrees (8, 14, 35).

In 12 pedigrees, the inheritance is not clearly defined (pedigrees 1, 4, 5, 9, 10, 11, 13, 18, 19, 30, 34, 36).

In one of the pedigrees, X-chromosomal dominant or AD trait was observed (SI 2 pedigree 28), and in one, X-chromosomal inheritance was suspected (SI 2 pedigree 23).

The pedigrees with VWS (SI 2 pedigree 26, 29, 41) had 80% penetrance in three successive generations, indicating a substantial AD inheritance. The patients with Stickler syndrome (pedigree 24) (three patients, two of them were siblings) presented AD inheritance with variable expressivity. The patients with Nager syndrome (pedigree 22) were siblings and affected by either AD or AR traits. One family member of these pedigrees had facial and craniofacial dysmorphic characteristics (pedigree 42). Although a genetic test was not performed, clinical phenotypes in the other family members point to this inheritance mode.

## Discussion

This study presents the clinical characterization and the inheritance patterns of patients with CL/P (SI 2) examined at Charité - Universitätsmedizin Berlin between 28.01.1999 and 25.05.2000. Retrospective studies, like this one, imply limitations related to selection bias, specific environmental risk exposures, and other confoundings associated with small population studies. Nevertheless, it is a well-documented database due to the interdisciplinary approach, the extensive geographic location of patients’ origin, and the patients’ and family members’ long-term follow-up.

In this study, only 50% of the center’s patients were included, and almost 50% of them were willing to participate. Nevertheless, we were able to examine and interview 1257 of their relatives and construct 42 pedigrees. Associated anomalies and syndromes were recorded. Data on stillbirths or early deaths are not presented in the study. Genome-wide linkage analysis has already been performed for pedigrees 6 and 10 (SI 2) [11].

### Cleft type, sidedness, and laterality

Individuals with UCL/P represent 65.7% of the index patients of this study (Table 1)*.* Left-sided CL/P is the most common type, affecting 2/3 of all patients with UCL/P [18]*.*

The offspring’s cleft laterality is not consistent with that of the affected parent [19, 20].

Hagberg et al. reported a high incidence of unilateral clefts (87.6%), and only 12.4% was the bilateral involvement [21]. A Norwegian population-based study, with data collected for more than 30 years [22], comprised almost 40% of patients with CL/P, 34% CPO, and 15% BCLP [22]. In this study, patients with CLP represent the biggest group of index patients (38.2%), followed by the group of patients with CPO (25.2%) and BCLP (19.5%) (Table 1). Facial clefts count only for 0.43–0.73% of all OFC [23], which agrees with our findings, where only one patient was registered (0.4%).

### Associated congenital malformations

A broad range (3% to 72%) of patients with OFC and associated malformations of other organs has been reported [7]. The prevalence of the associated anomalies is closely related to the severity of the cleft [20]. Patients with CPO have a higher occurrence of associated malformations compared to patients with CLP [5, 23, 24], which is a common finding with this study. The individuals with BCLP are more commonly affected with malformations and syndromes than the UCL/P patients [25]. Almost 1/3 of the associated malformations were observed in the skeletal system and extremities [26]. Contrarily, other studies reported the head and neck [7] or the face [5] as the most common areas with associated anomalies. The association of CL/P with cardiovascular anomalies is varying widely, ranging from 4.3 [27] to 63.4% [7]. Abnormalities of the CNS (14.3%), the urogenital system [5], other facial (13.0%), and miscellaneous malformations (4.1%) [26] have also been reported.

The studies’ high variability is due to the examination criteria, type of cleft, malformations included, age of the patient at the diagnosis, and ethnic background [28].

Furthermore, some anomalies or syndromes are not detectable in the first year of life (e.g., lip pits in the VWS). Therefore, the clinical diagnosis in the absence of known family history is challenging. Due to the developmental variations, almost only 12% of the anomalies are detected between the 1st and 6th years of age [29].

### Associated syndromes

Almost 70% of the patients with a cleft are nonsyndromic [3]. In the other 30%, more than 600 syndromes have been identified. The majority of the syndromes have been observed in the CPO clinical phenotype [9, 30]. Nevertheless, the genetic and environmental triggers of syndromic clefts’ pathogenesis can be the same as those of nonsyndromic (nsCL/P) [31]. The most common syndromes observed among the index patients were the PRS (7 patients) and the VWS (5 patients) (Table 4). Comparing these findings with other studies, the most common syndromes involving OFC are the VWS (in 2% of the CL/P patients) [32], the 22q11.2 deletion, the Kallmann syndrome, and the PRS [33].

### Sex distribution according to cleft type and associated malformations

In this study, the overall sex distribution follows other studies [27] showing a predominance in male individuals (Table 1). In a multivariable analysis, the males had a 2.5-fold higher OFC chance (OR = 2.39; 95%) [34]. Nevertheless, there is variability in sex distribution according to the cleft type (Table 1). Therefore, following other studies, there is a male predisposition in CL and CLP patients, with a male to female ratio of 1.75:1 [35]. Among the patients with BCLP, males were twice as much as females [36]. Nonetheless, the incidence of patients with CPO is higher in females [37], a common finding with this study.

Females were more likely (up to 62%) to have a severe type of cleft and associated congenital abnormalities [22].

### Inheritance and genetics

OFC has one of the highest rates of family recurrence [38]. The heritability in twins and singletons was 90%, and only 10% was probably attributed to environmental factors [39]. CLP risk among siblings is 30 times higher than the prevalence of an average population [40].

Almost 25% [41] of the patients with a positive family history have CL/P [21] and 12% CPO [41, 42]. In a longitudinal population-based study, patients with CPO have a higher risk of recurrence in first-degree relatives [19]. In this study, a lower occurrence has been registered (Table 1).

We recognized that CPO was observed in two families of affected mothers. Nevertheless, other studies reported that the recurrent risk was not associated, either with the sex of the primary patient or the severity and sidedness of the cleft [42].

**Epigenetic factors** [43] or genes that contribute to organ development’s laterality may define the laterality of the cleft, e.g., the BCOR-gene in the oculo-facio-cardio-dental syndrome (OFCD) [44, 45]. Furthermore, there is no common polygenic association of nonsyndromic CL/P and CPO [46].

The genetic basis of nsCL/P is complex, and until now, not well understood [47]. So far, more than 50 genes have been identified for the pathogenesis of nsCL/P [48] and more than 260 for the syndromic phenotypes [49].

Nevertheless, the maternal genes without environmental exposures or the synergy with fetal genes are not responsible for the pathogenesis and the phenotypic variability of patients with CL/P [50]. Maternal risk factors and teratogens may trigger the pathogenic mechanism of nsCL/P [11, 51].

Variants of the interferon regulatory factor 6 (*IRF6*) gene account for 12% of the genetic pathogenesis of nsCL/P [52, 53]. Mutations in *PBX1*, *PBX2*, and *TP63* have contributed to nsCL/P’s pathogenesis [48] and in *TBX22*, *P63*, and *FGFR1* to sCL/P [53]. The X-chromosomal recessive form has been identified in families with a cleft of the secondary palate (overt or submucous), and it is often associated with ankyloglossia [54].

A gene-specific mutation or the associated *IRF6* haplotype could raise the recurrent familial risk from the practical value of 3 to 5%, making critical the genetic counseling for subsequent pregnancy [53]. Therefore, molecular preventing counseling in families at risk and studies to identify exogenous risk factors are mandatory. The cleft team members should be suspicious of the co-occurrence of associated malformations. Early genetic counseling and regular follow-up appointments are required [55].

## Conclusion

Positive family history was observed in 23.9% of the index patients and association with a syndrome in 9.2%. Patients with CPO had more frequently an associated syndrome than patients with other cleft phenotypes. Skeletal (27.7%) and eye deformities (22.9%) were the most common associated malformations in this study. Male patients were more commonly afflicted (71.4%) with CL/P and females (59.7%) with CPO phenotypes. The overall prevalence of individuals with CL/P and their pedigrees with associated malformations and syndromes emphasizes the need for early identification, interdisciplinary interaction, and long-term planning. Phenotypic variability in pedigrees makes the understanding of etiopathogenesis more difficult. These pedigrees did not propose a particular inheritance trait, but it has been produced data for further molecular analysis.

## Supplementary Information

ESM 1 (DOCX 23.5 kb)

ESM 2 (DOCX 3.32 mb)
