# Supplementary material for: Clinical characterization of 266 patients and family members with cleft lip and/or palate with associated malformations and syndromes
Source: Clin Oral Investig. 2021 Mar 24;25(9):5531–40. doi: 10.1007/s00784-021-03863-2 (PMC8370934; doi:10.1007/s00784-021-03863-2)
Supplement: Supplementary file 1 — (DOCX 23.5 kb) [file 784_2021_3863_MOESM1_ESM.docx]

Article title: **Clinical characterization of 266 patients and family members with cleft lip and/or palate with associated malformations and syndromes**

Journal name: Clin Oral Invest

Author names: **Theodosia Bartzela^1^, Björn Theuerkauf^2^, Elisabeth Reichardt^3^, Malte Spielmann^4,5^, Charlotte Opitz^1^**

**Corresponding author:**

Theodosia Bartzela

Charité - Universitätsmedizin Berlin, CC3

Institute of Dental and Craniofacial Sciences

Dept. of Orthodontics, Dentofacial Orthopedics and Pedodontics

Aßmannshauser Str. 4-6, D-14197 Berlin

E-Mail: theodosia.bartzela@charite.de

**Supplementary information 1**

**SI 1** The medical and family history of the patients was supplemented by a standardized questionnaire used by the Department of Human Genetics of Charité-Universitaetsmedizin, which was adapted to the needs of the study).

**Personal details**

Family Name, Name

Sex

Date of Birth

Address, telephone number

**Medical history**

Type of cleft

Associated anomaly (Ear, eyes, lips, mouth, heart, skeleton etc.)

Disease (Ear, eyes, lips, mouth, heart, skeleton etc.)

Syndrome

**Family history**

Relatives with:

1. orofacial cleft
2. other associated anomalies
3. abortions, early births, stillbirths or deaths
