# Supplementary material for: Clinical characterization of 266 patients and family members with cleft lip and/or palate with associated malformations and syndromes
Source: Clin Oral Investig. 2021 Mar 24;25(9):5531–40. doi: 10.1007/s00784-021-03863-2 (PMC8370934; doi:10.1007/s00784-021-03863-2)
Supplement: Supplementary file 2 — (DOCX 3.32 mb) [file 784_2021_3863_MOESM2_ESM.docx]

Article title: **Clinical characterization of 266 patients and family members with cleft lip and/or palate with associated malformations and syndromes**

Journal name: Clin Oral Invest

Author names: **Theodosia Bartzela^1^, Björn Theuerkauf^2^, Elisabeth Reichardt^3^, Malte Spielmann^4,5^, Charlotte Opitz^1^**

**Corresponding author:**

Theodosia Bartzela

Charité - Universitätsmedizin Berlin, CC3

Institute of Dental and Craniofacial Sciences

Dept. of Orthodontics, Dentofacial Orthopedics and Pedodontics

Aßmannshauser Str. 4-6, D-14197 Berlin

E-Mail: theodosia.bartzela@charite.de

**Supplementary information 2**.

42 pedigrees of patients with orofacial cleft. Type of cleft, laterality, associated anomalies, microsymptomy, and deaths shortly after birth or still births of affected individuals have been indicated.

Male, female, unknown sex, index patient,



right, left and bilateral cleft lip (CL) and/or alveolus (CL/A)

right, left and bilateral cleft lip and palate (CLP)

Cleft lip and palate (CPO) or in soft palate only (CSO)

Microsymptoms



† Associated anomaly or syndrome

_NND, SB._ Death shortly after birth

stillbirth

evn.: eventually

﻿A question mark (?) indicates an unknown phenotype (pedigree 42)


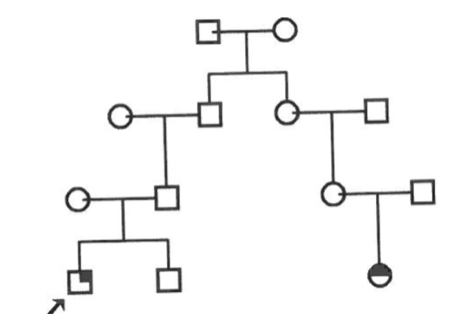


**pedigree 1**

Index patient: right CLA, cousin: bilateral CL

Mode of inheritance: not defined


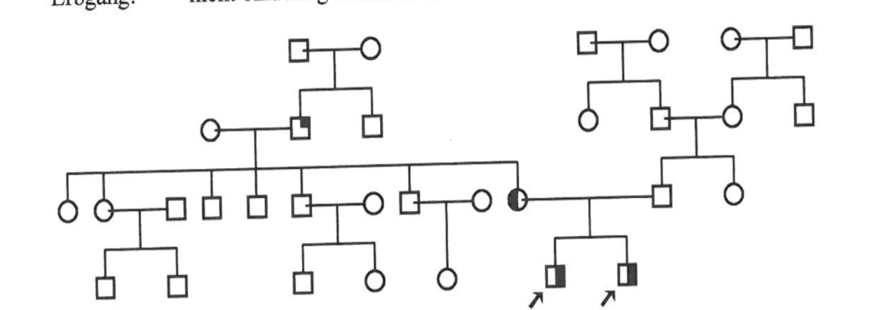


**pedigree 2**

Index patients: right CLP (CLP), mother: left CLP, grandfather: right CLA

Mode of inheritance: autosomal dominant


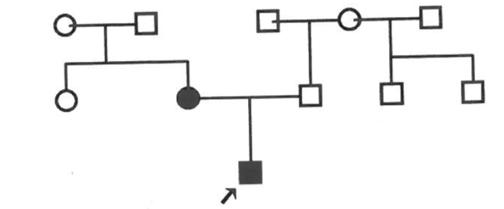


**pedigree 3**

Index patient: bilateral CLP, mother: bilateral CLP

Mode of inheritance: evt. autosomal dominant


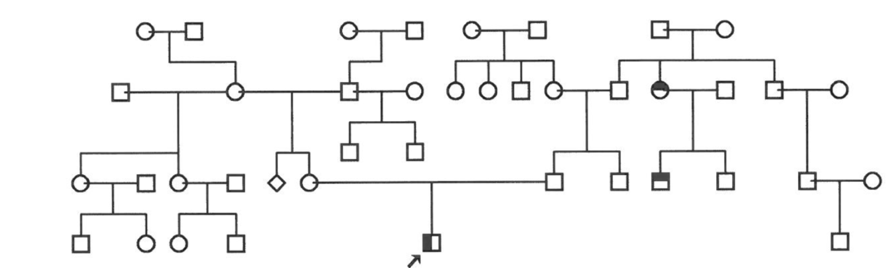


**pedigree 4**

Index patient: left CLA and uvula bifida, brother of the grandfather: bilateral CLA, granduncle: bilateral CLA

Mode of inheritance: not clearly defined


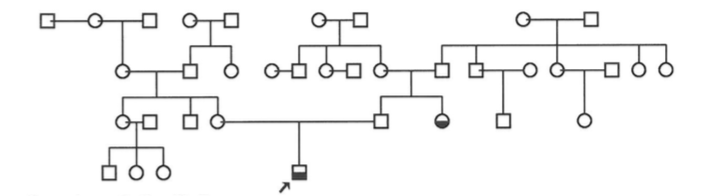


**pedigree 5**

Index patient: CPO, aunt: CPO

Mode of inheritance: not clearly defined


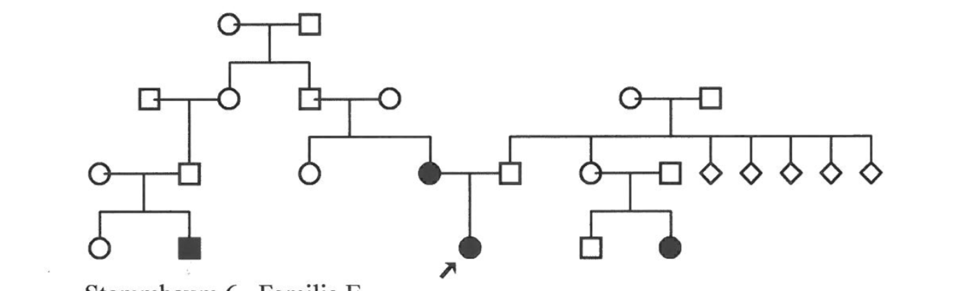


**pedigree 6**

Index patient: bilateral CLP, mother: bilateral CLP, grand cousin: bilateral CLP

Mode of inheritance: evt. autosomal dominant


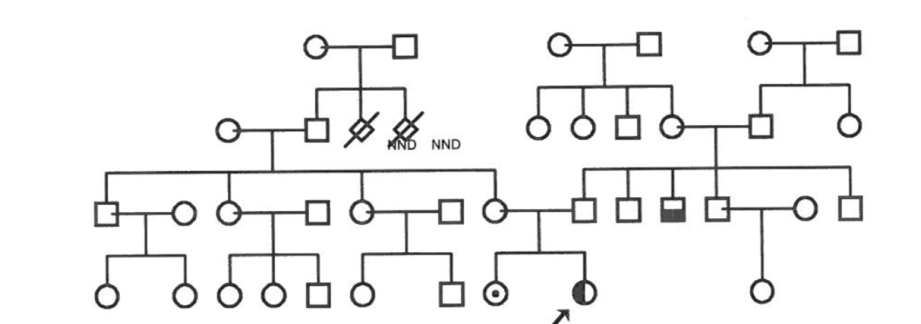


**pedigree 7**

Index patient: left CLP, uncle: CPO, sister: agenesis of 12, 22

Mode of inheritance: evt. autosomal dominant with reduced penetrance


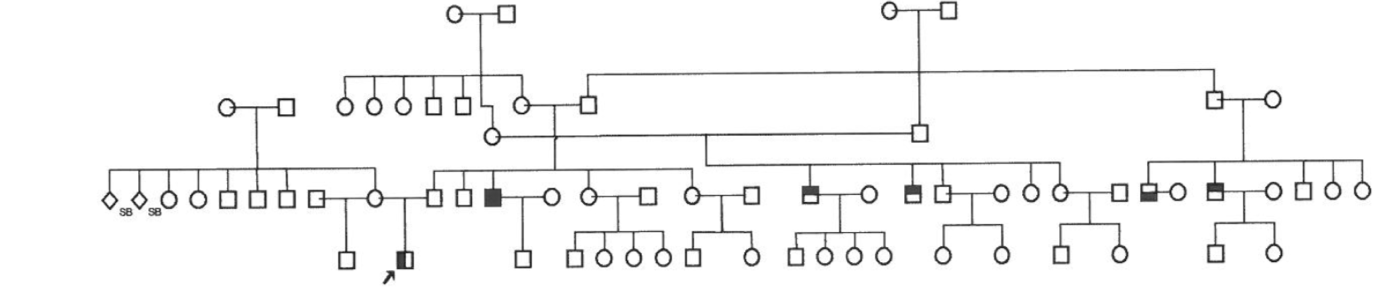


**pedigree 8**

Index patient: left CLP, Uncle: bilateral CLP, grand cousin: bilateral CL, cousin: bilateral CL, cousin: CPO, cousin: bilateral CLA

Mode of inheritance: autosomal recessive (mainly only one generation)


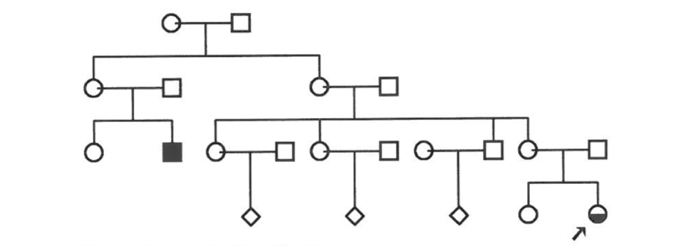


**pedigree 9**

Index patient: CPO, grand uncle: CLP

Mode of inheritance: not clear, sporadic also possible


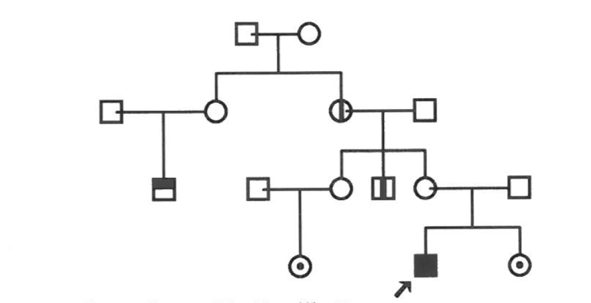


**pedigree 10**

Index patient: bilateral CLP, grand uncle: bilateral CLA, sister: agenesis of 12, cousin: glossoptosis, uncle and grandmother: additional finger

Mode of inheritance: not clearly defined


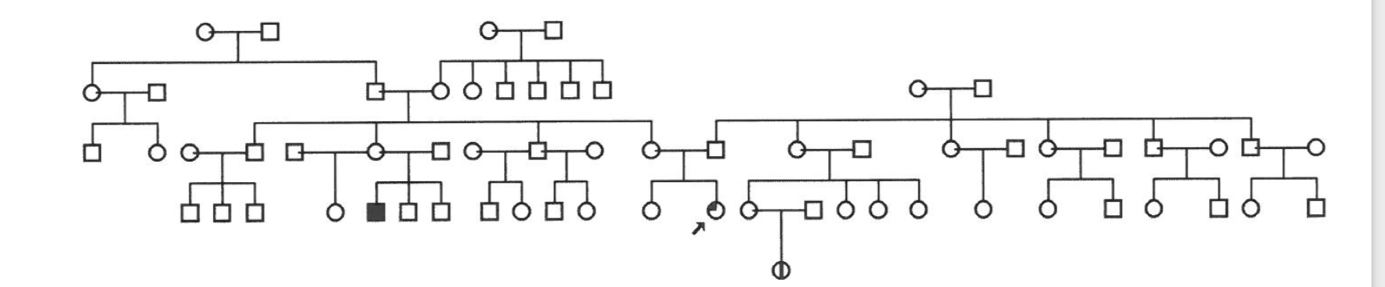


**pedigree 11**

Index patient: left CLA, cousin: CLP, grand nice: clubfoot

Mode of inheritance: evt. autosomal recessive


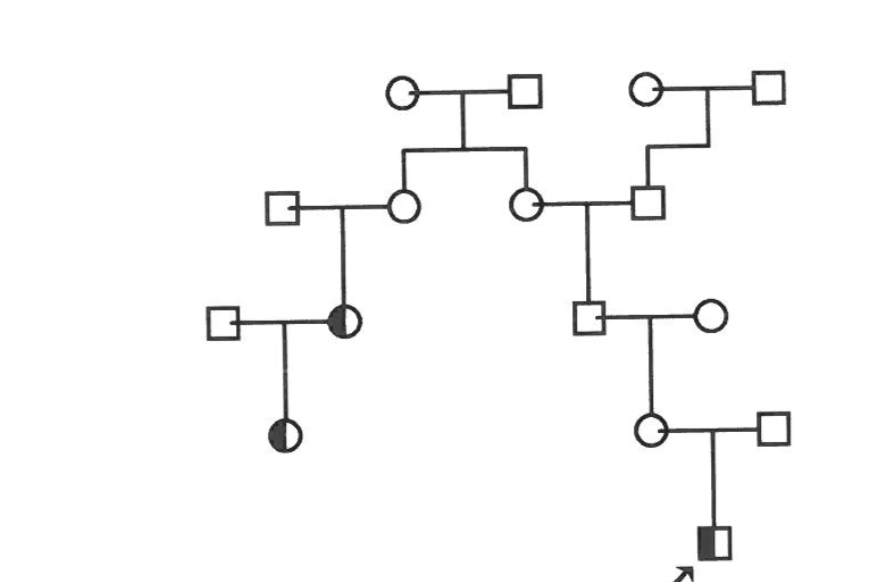


**pedigree 12**

Index patient: left CLP, grant aunt and her mother: left CLP

Mode of inheritance: evt. autosomal dominant with incomplete penetrance (same sidedness and type of cleft)


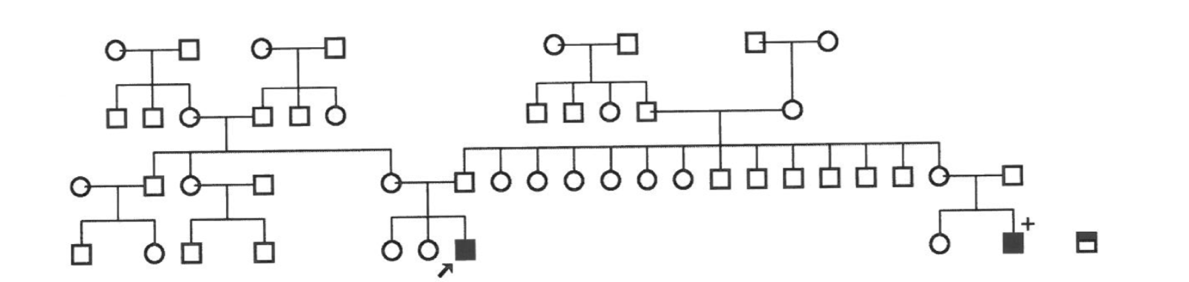


**pedigree 13**

Index patient: bilateral CLP, cousin: CPO, midfacial cleft, blind, grand cousin: bilateral CLA

Mode of inheritance: evt. autosomal recessive


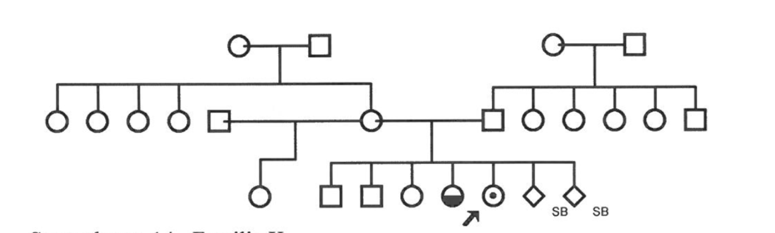


**pedigree 14**

Index patient: submucosal CPO, sister: CPO,

Mode of inheritance: evn. autosomal recessive


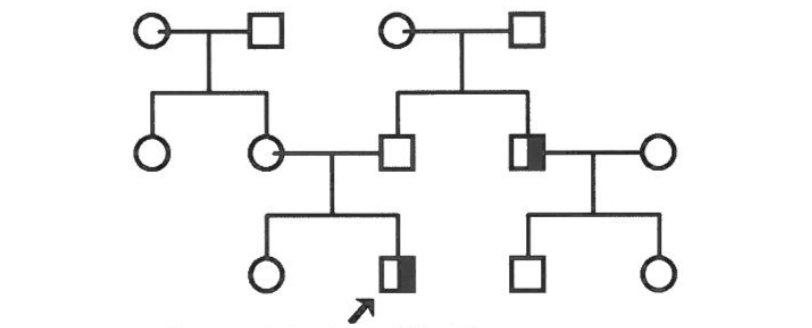


**pedigree 15**

Index patient: right CLP, uncle: right CLP

Mode of inheritance: evt. autosomal dominant with reduced penetrance (not X-linked)


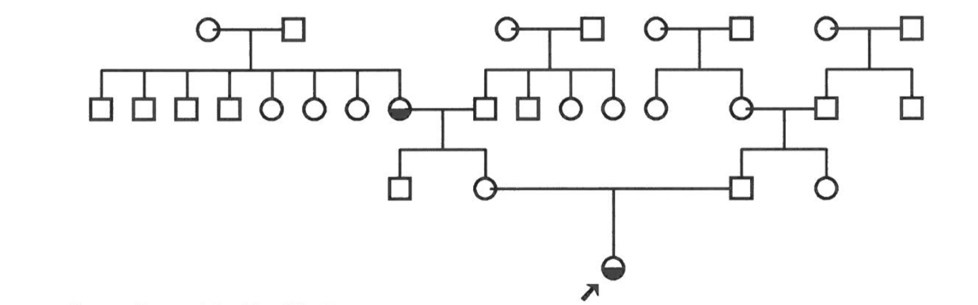


**pedigree 16**

Index patient: CPO, grandmother: CPO

Mode of inheritance: autosomal dominant with reduced penetrance


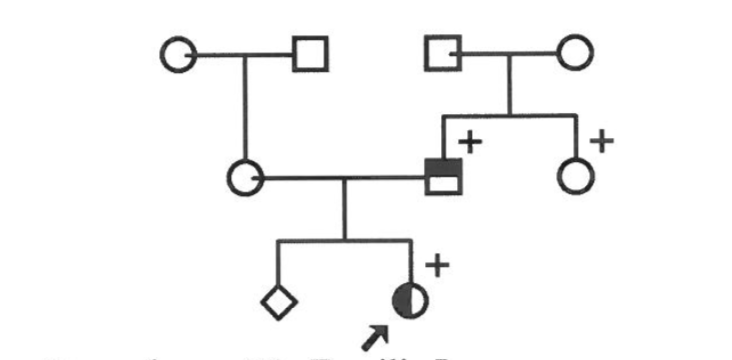


**pedigree 17**

Index patient: left CL and CSO and ventricle septal defect, father: bilateral CLA and ventricle septal defect

Mode of inheritance: indicate an autosomal dominant inheritance


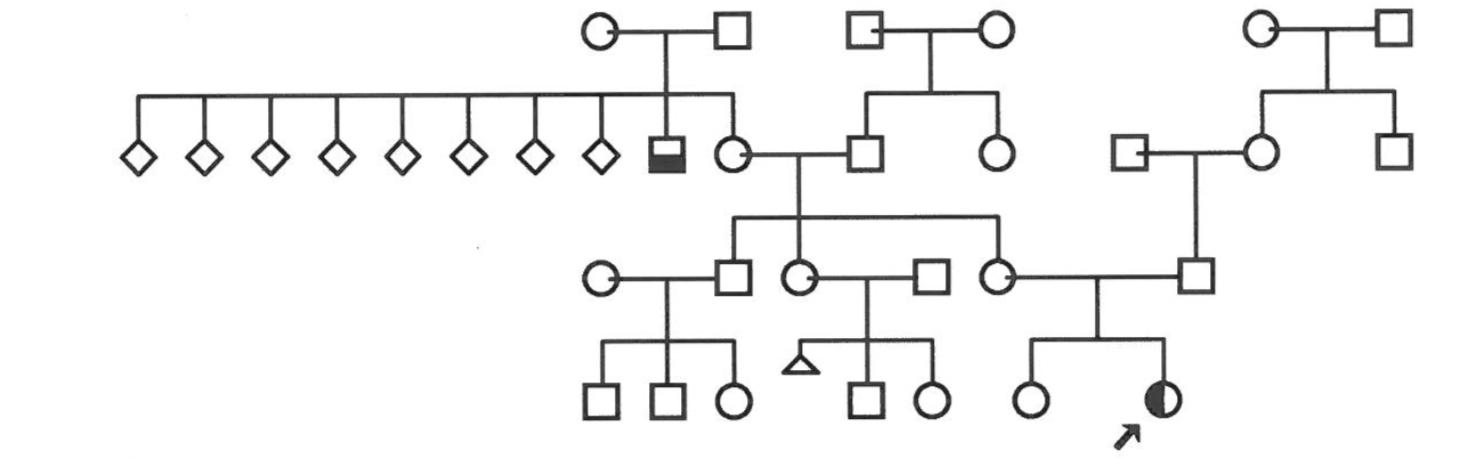


**pedigree 18**

Index patient: left CLP, brother of grandmother: CPO

Mode of inheritance: not clearly defined


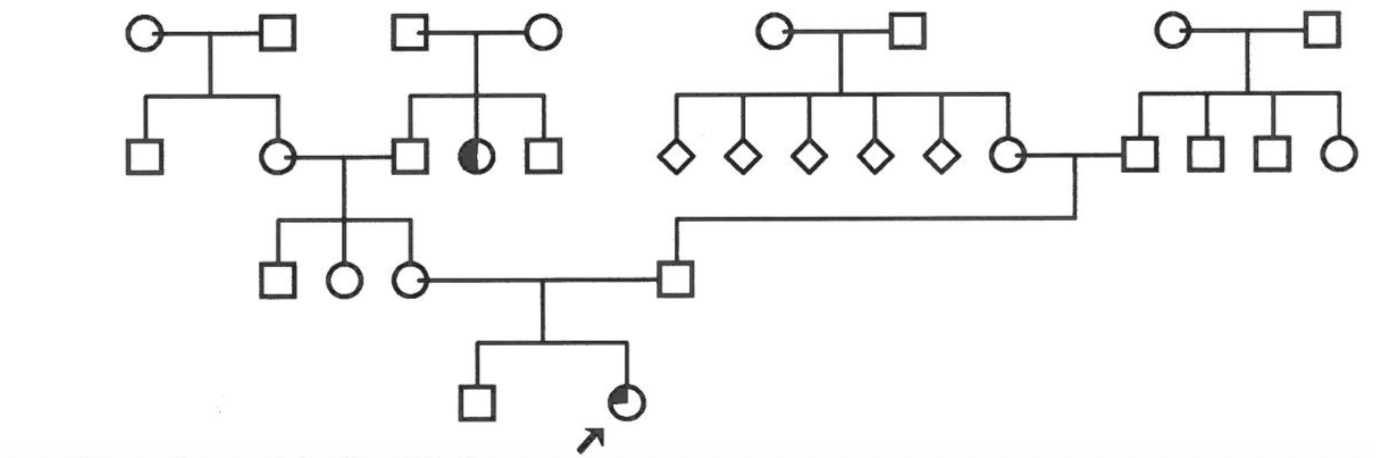


**pedigree 19**

Index patient: left CLA, sister of grandmother: left CLP

Mode of inheritance: not clearly defined


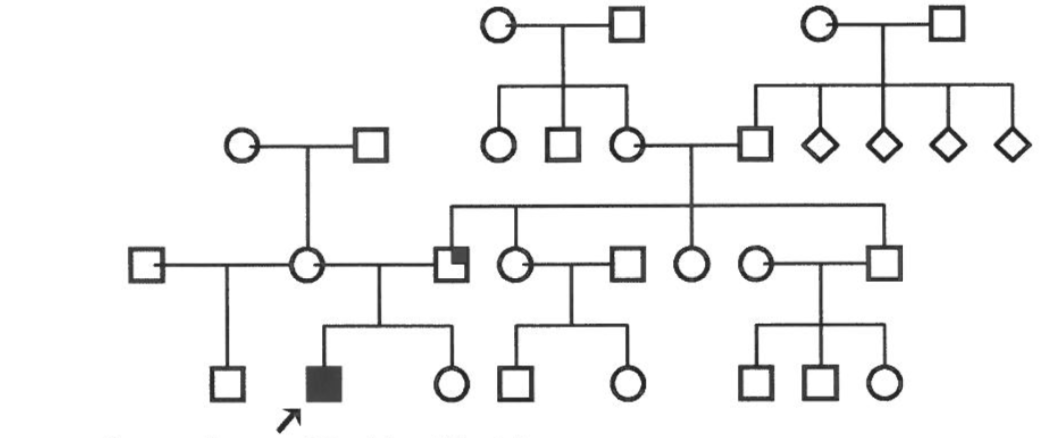


**pedigree 20**

Index patient: bilateral CLP, father: right CLA

Mode of inheritance: evt. autosomal dominant


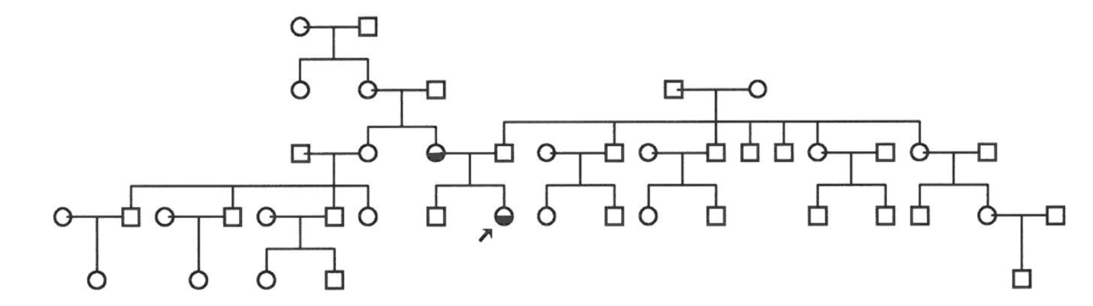


**pedigree 21**

Index patient: CPO, mother: CPO

Mode of inheritance: evt. autosomal dominant


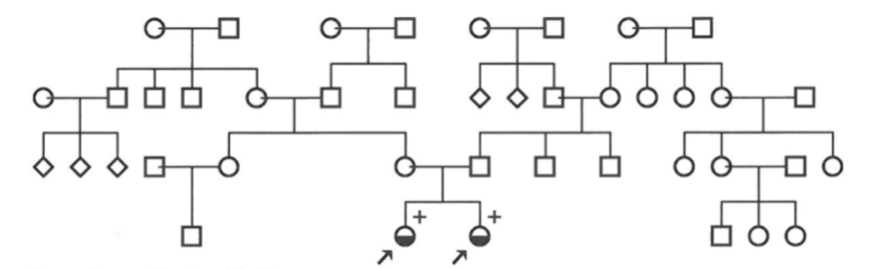


**pedigree 22**

Index patients: Nager syndrome, CPO

Mode of inheritance: evt. autosomal dominant or recessive (sisters)


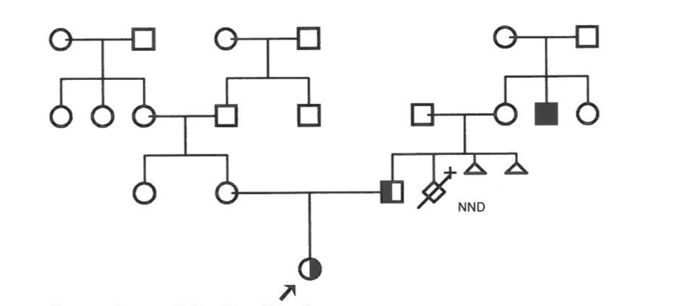


**pedigree 23**

Index patient: right CLP, father: left CLP, brother of grandmother: bilateral CLP

Mode of inheritance: autosomal dominant


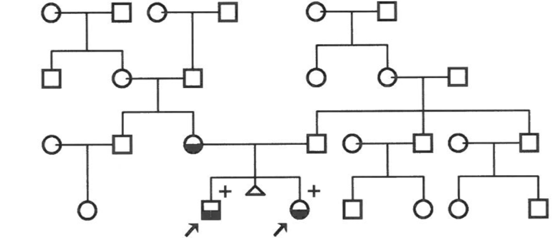


**pedigree 24**

Index patients: Stickler syndrome, CPO, mother: Stickler syndrome, CPO

Mode of inheritance: autosomal dominant with different degree of variable expressivity


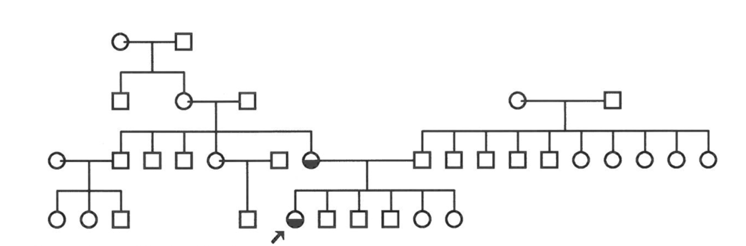


**pedigree 25**

Index patient: CPO, mother: CPO

Mode of inheritance: evt. autosomal dominance


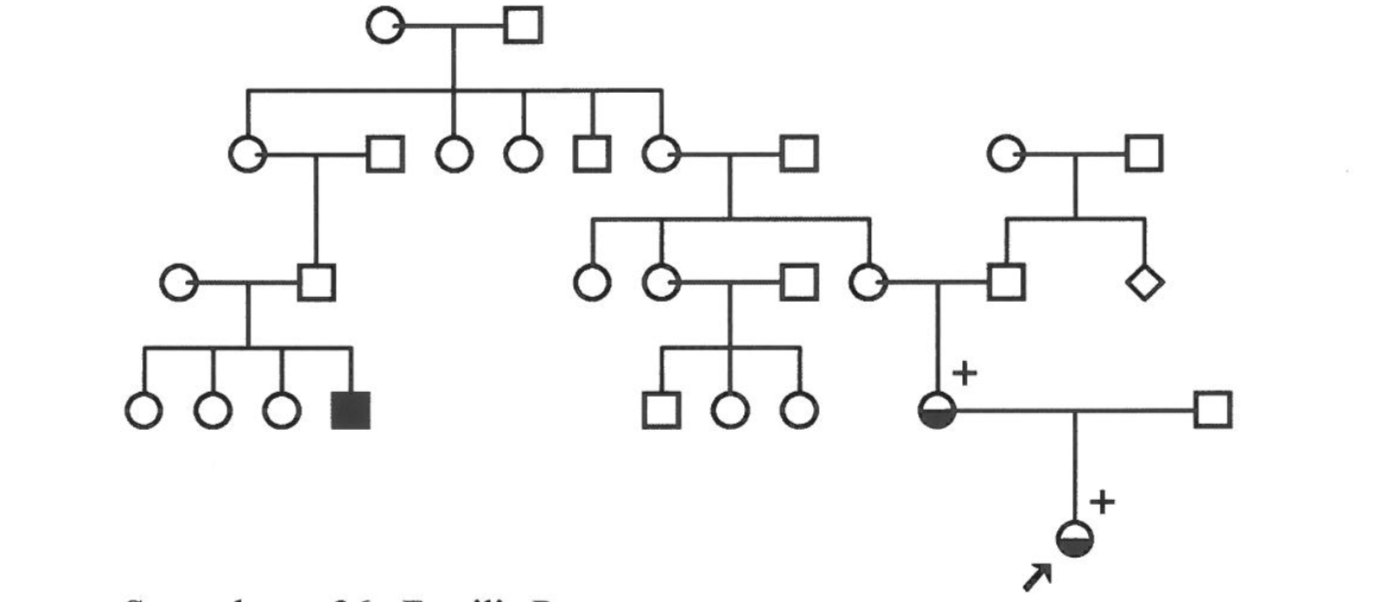


**pedigree 26**

Index patient: van der Woude syndrome, CPO, mother: van der Woude syndrome, CPO, grand uncle: bilateral cCLP. Cleft or symptoms of van der Woude syndrome in the grandmother and grand aunt of the mother’s side

Mode of inheritance: van der Woude syndrome has autosomal dominant inheritance with about 80% penetrance


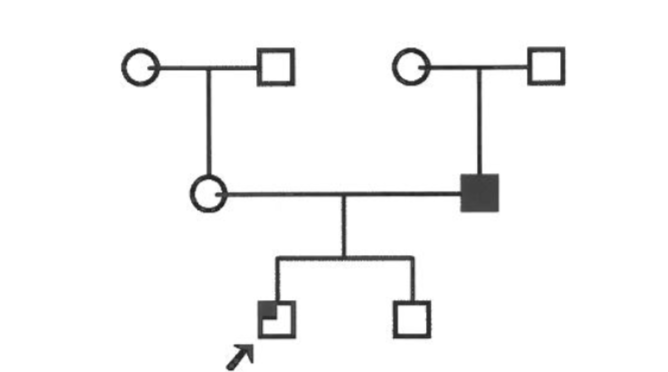


**pedigree 27**

Index patient: left CL, father: bilateral CLP

Mode of inheritance: evt. autosomal dominant


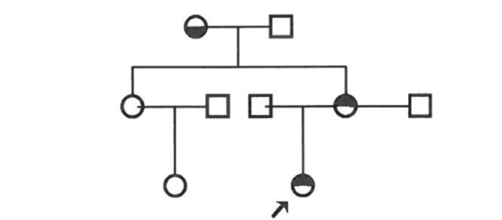


**pedigree 28**

Index patient: left CL, father: bilateral CLP

Mode of inheritance: evt. autosomal dominant


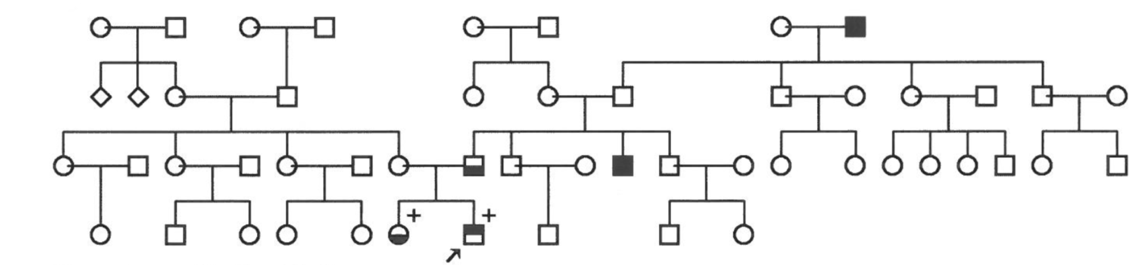


**pedigree 29**

Index patient: bilateral CLA and van der Woude syndrome, sister: CPO, father: CPO, uncle: CLP,

great grandfather: CLP

Mode of inheritance: van der Woude syndrome: autosomal dominant with 80% penetrance


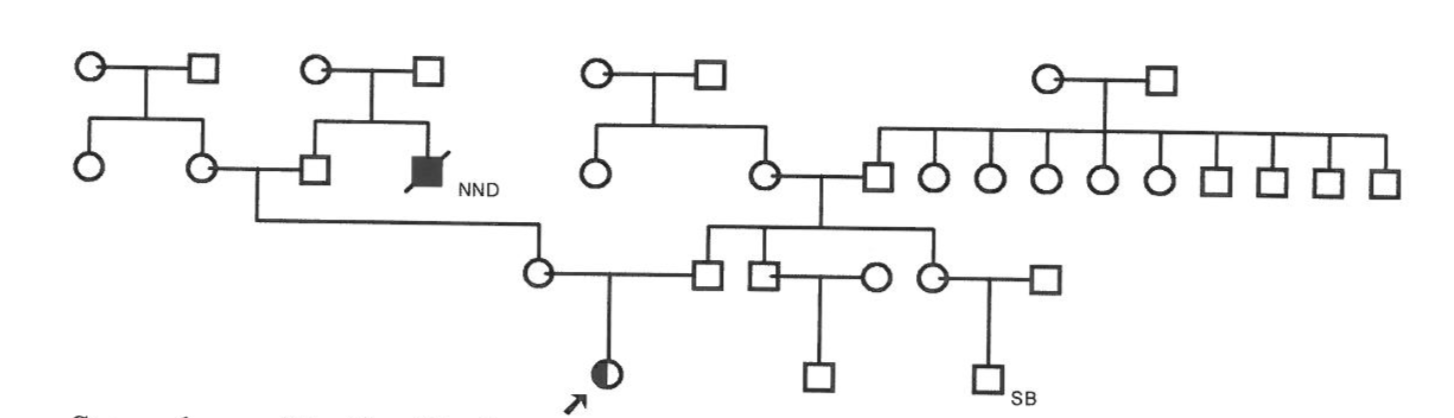


**pedigree 30**

Index patient: left CLP, brother of the grandfather: facial cleft, dead shortly after birth

Mode of inheritance: not clearly defined


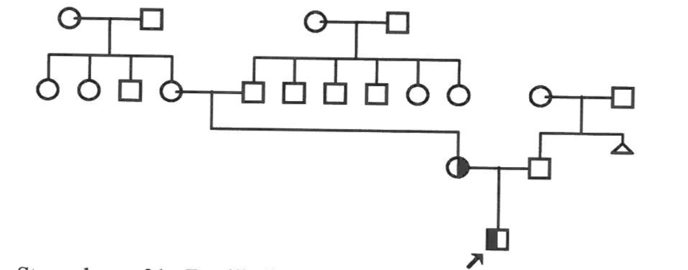


**pedigree 31**

Index patient: left CLP, mother: right CLP,

Mode of inheritance: evt. autosomal dominant


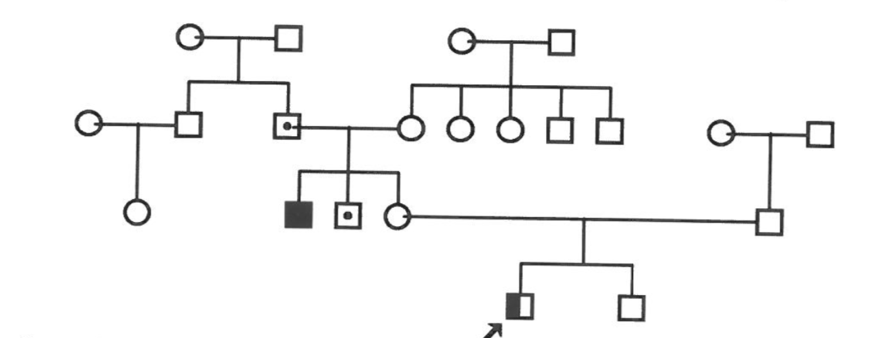


**pedigree 32**

Index patient: left CLP, uncle: bilateral CLP, uncle and grandfather: perforation of the nasal septum

Mode of inheritance: evt. autosomal dominant


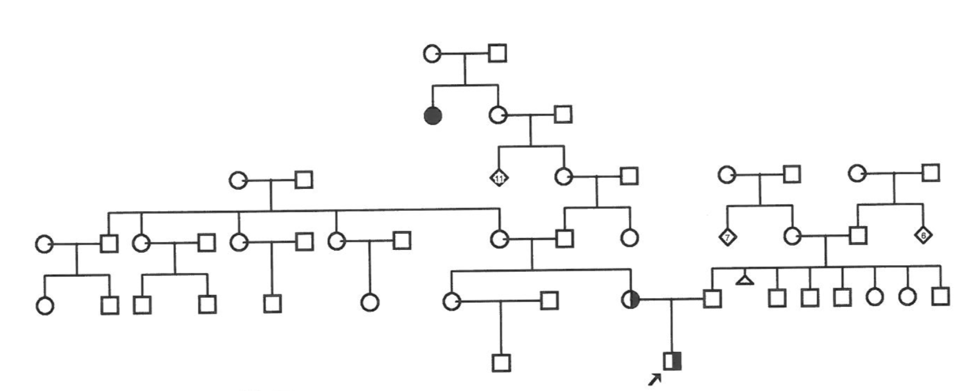


**pedigree 33**

Index patient: right CLP, right CLP, grand grandmother: bilateral CLP

Mode of inheritance: evt. autosomal dominant with reduced penetrance


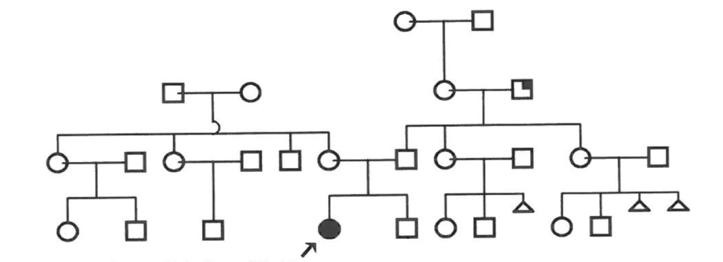


**pedigree 34**

Index patient: bilateral CLP, grandfather right CL(A?)

Mode of inheritance: not clearly defined


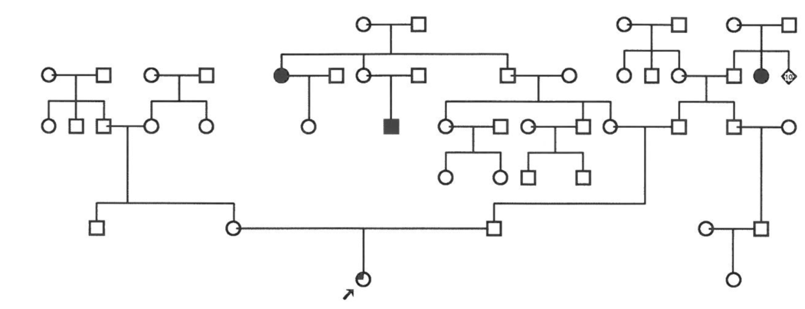


**pedigree 35**

Index patient: left CLA, grand uncle: CLP, sister of the grand grandfather: CLP

Mode of inheritance: evt. autosomal dominant


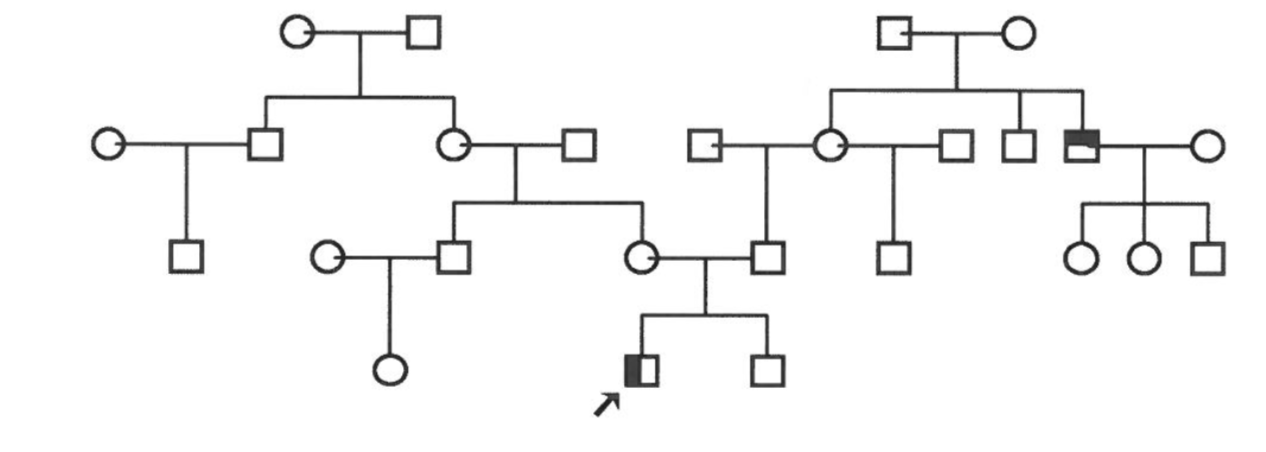


**pedigree 36**

Index patient: left CLP, brother of the grandmother: bilateral CL

Mode of inheritance: not clearly defined


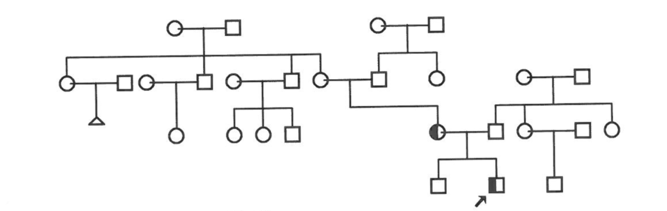


**pedigree 37**

Index patient: left CLP, mother: left CLP

Mode of inheritance: evt. autosomal dominant


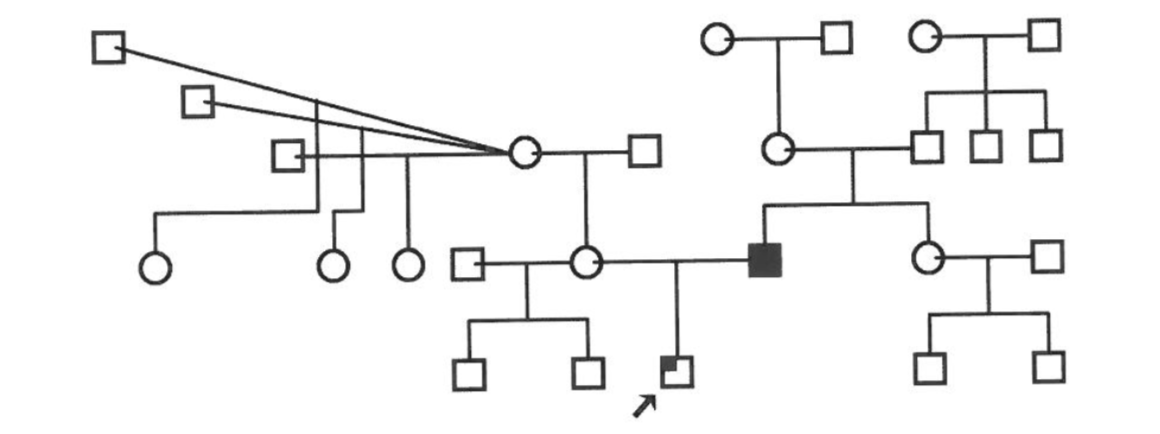


**pedigree 38**

Index patient: left CLA, father: bilateral CLP

Mode of inheritance: evt. autosomal dominant


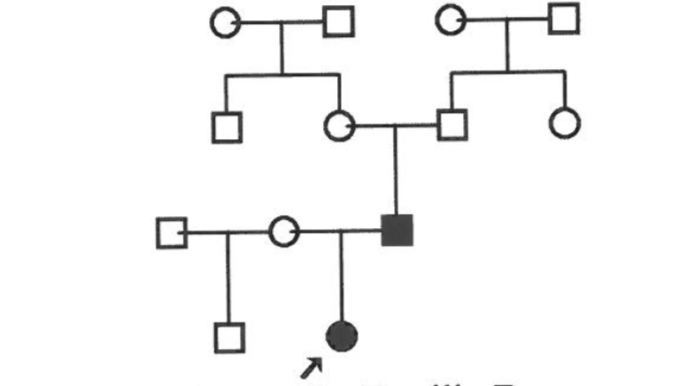


**pedigree 39**

Index patient: bilateral CLP, father: bilateral CLP

Mode of inheritance: evt. autosomal dominant


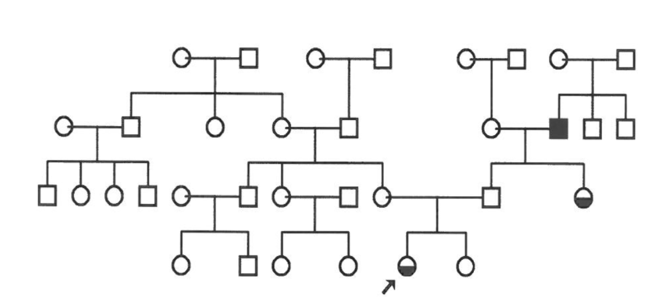


**pedigree 40**

Index patient: CSO, aunt: CPO, grandfather: type of cleft unknown

Mode of inheritance: autosomal dominant with reduced penetrance


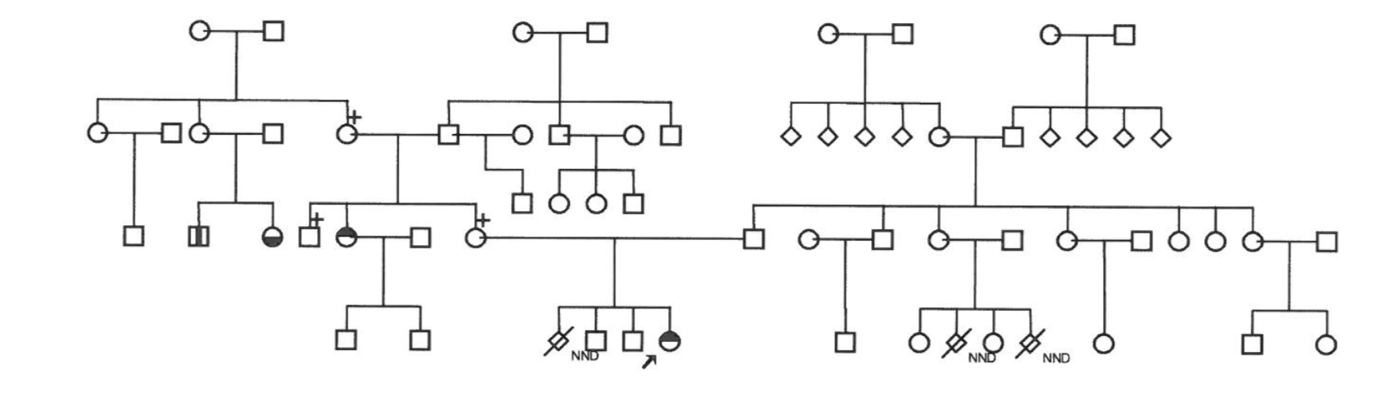


**pedigree 41**

Index patient: van der Woude with CSO, aunt: CPO, grand aunt: CPO, mother, grandmother, uncle: epilepsy, grand uncle: severely disabled

Mode of inheritance: van der Woude syndrome: autosomal dominant with 80% penetrance


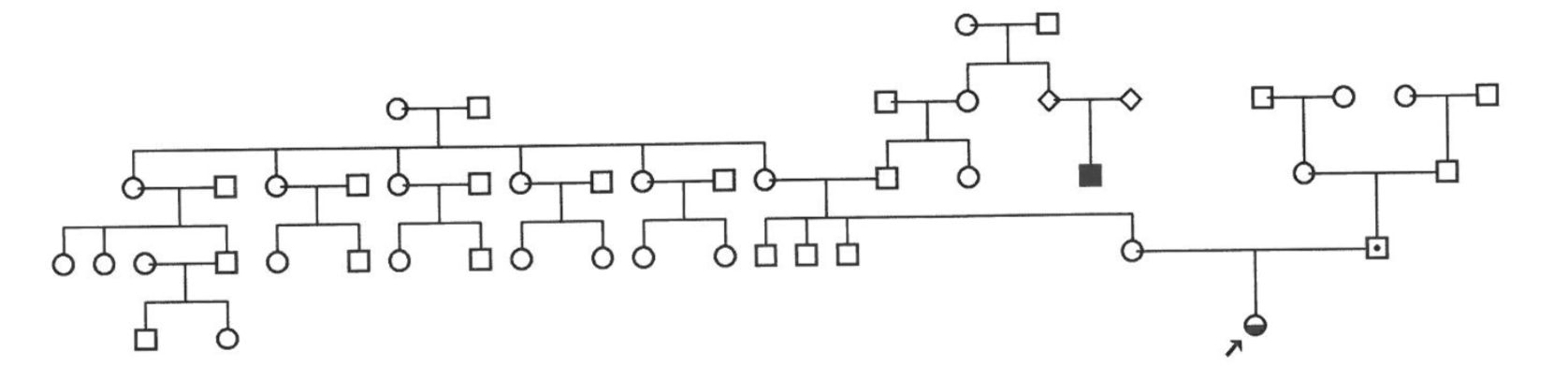


**pedigree 42**

Index patient: cranio-facial dysmorphia syndrome, CPO, father: uvula bifida, grand uncle: CLP?

Mode of inheritance: evt. autosomal dominant
